# Upregulation of Phosphorylated HSP27, PRDX2, GRP75, GRP78 and GRP94 in Acquired Middle Ear Cholesteatoma Growth

**DOI:** 10.3390/ijms140714439

**Published:** 2013-07-11

**Authors:** Kuen Yao Ho, Tai Sheng Yeh, Han Hsiang Huang, Kuo Feng Hung, Chee Yin Chai, Wan Tzu Chen, Shih Meng Tsai, Ning Chia Chang, Chen Yu Chien, Hsun Mo Wang, Yu Jen Wu

**Affiliations:** 1 Department of Otorhinolaryngology, Kaohsiung Medical University Hospital, Kaohsiung 80756, Taiwan; E-Mails: kuyaho@kmu.edu.tw (K.Y.H.); chenyu@cc.kmu.edu.tw (C.Y.C.); 2 Department of Otorhinolaryngology, School of Medicine, College of Medicine, Kaohsiung Medical University, Kaohsiung 80756, Taiwan; 3 Department of Food Science and Nutrition, Meiho University, Pingtung 91202, Taiwan; E-Mail: x00010091@meiho.edu.tw; 4 Department of Beauty Science, Meiho University, Pingtung 91202, Taiwan; E-Mail: hhuang.adsl@msa.hinet.net; 5 Graduate Institute of Applied Health and Biotechnology, Meiho University, Pingtung 91202, Taiwan; E-Mail: q87634@yahoo.com.tw; 6 Department of Pathology, Kaohsiung Medical University Hospital, Kaohsiung 80756, Taiwan; E-Mails: cychai@cc.kmu.edu.tw (C.Y.C.); wanzi@cc.kmu.edu.tw (W.T.C.); 7 Department of Pathology, School of Medicine, College of Medicine, Kaohsiung Medical University, Kaohsiung 80756, Taiwan; 8 Department of Public Health, School of Medicine, College of Medicine, Kaohsiung Medical University, Kaohsiung 80756, Taiwan; E-Mail: tsaism@kmu.edu.tw; 9 Department of Preventive Medicine, Kaohsiung Medical University Hospital, Kaohsiung 80756, Taiwan; E-Mail: aka@mail.kmuh.org.tw; 10 Department of Otorhinolaryngology, Kaohsiung Municipal Ta-Tung Hospital, Kaohsiung Medical University, Kaohsiung 80756, Taiwan; E-Mail: hmwang@cc.kmu.edu.tw

**Keywords:** cholesteatoma, HSP27, PRDX2, GRP75, GRP78, GRP94

## Abstract

Cholesteatoma is a destructive and expanding growth of keratinizing squamous epithelium in the middle ear or petrous apex. The molecular and cellular processes of the pathogenesis of acquired middle ear cholesteatoma have not been fully understood. In this study, comparative proteomic analysis was conducted to investigate the roles of specific proteins in the pathways regarding keratinocyte proliferation in cholesteatoma. The differential proteins were detected by comparing the two-dimension electrophoresis (2-DE) maps of the epithelial tissues of 12 attic cholesteatomas with those of retroauricular skins. There were 14 upregulated proteins in the epithelial tissues of cholesteatoma in comparison with retroauricular skin. The modulation of five crucial proteins, HSP27, PRDX2, GRP75, GRP78 and GRP94, was further determined by RT-PCR, Western blot and immunohistochemistry. Phosphorylation of HSP27 at Ser-82 was identified by mass spectroscopy. The results of this study suggested that phosphorylated HSP27 is the end expression of two potential signal-transduction pathways, and together with PRDX2, they are very likely involved in the proliferation of keratinocytes in cholesteatoma. Upregulations of GRP75, GRP78 and GRP94 in keratinocytes may be able to counter endoplasmic reticulum stress, to inhibit cell apoptosis, to prevent protein unfolding and to promote cholesteatoma growth.

## 1. Introduction

Cholesteatoma is a destructive and expanding growth of keratinizing squamous epithelium in the middle ear cavity [1–5]. A perforation of the eardrum caused by chronic infection or direct trauma could lead to cholesteatoma. The skin over the outer surface of the eardrum could grow through the perforation and into the middle ear. Small remnants of skin of the eardrum (retraction pocket) are trapped into the middle ear in most patients [6]. Local infection leads to a disturbance of self-cleaning mechanisms. Cell debris and keratinocytes then accumulate inside the retraction pocket. Imbalance and vicious circles of epithelial proliferation, keratinocyte differentiation and maturation, prolonged apoptosis and disturbance of self-cleaning may occur. The inflammatory stimulus can induce an epithelial proliferation along with expression of lytic enzymes and cytokines [1–3]. Bacteria inside the retraction pocket produce some antigens, which are able to activate different cytokines and lytic enzymes [4,5]. Cholesteatoma keratinocytes undergo a change in behavior *in vivo* that is preserved after the cells are removed from the inflammatory environment of the middle ear [7]. Cholesteatoma could cause destruction of three ossicles located in the middle ear. It may result in hearing deterioration, deafness, physical imbalance and vertigo. Cholesteatoma has been recognized for decades as a destructive lesion of the skull base, which may erode and destroy important structure within the temporal bone. Its potentials for causing central nervous system complications, bone destruction and potential recurrence are key elements of the pathophysiology of cholesteatomas [8,9]. These features make it a potentially dangerous disease and difficult to treat. The etiopathogenesis of middle ear cholesteatoma is still controversial. It is possible that pathogenesis includes (1) the origin of keratinizing squamous epithelium; (2) a factor involved in the invasive and hyperproliferative behavior and (3) a first signal to start cholesteatoma development. As a result, it is a destructive process in the middle ear, resulting in erosion of surrounding bony structures.

The processes may involve some key proteins and pathways. It has been suggested that cholesteatoma is associated with activation of osteoclasts and a variety of mechanisms involving cellular functions. Identification of key proteins in the processes could provide important information for the treatment of the disease. The molecular and cellular processes of the pathogenesis of cholesteatoma have not been fully understood. Both autocrine and paracrine stimulations were shown to play important roles in the pathogenesis of cholesteatoma [10,11]. Growth factors, such as transforming growth factor α (TGF-α) and interleukin 1 (IL-1), were found to be responsible for hyperproliferation of keratinocytes in cholesteatoma [12,13]. Keratinocyte growth factor (KGF) has been shown to play a role in epithelial growth and differentiation in cholesteatoma [14]. Up-modulation of the KGF/KGF receptor (KGFR) has also been found in cholesteatoma, and its signaling could be also involved [14,15]. Investigation by Huisman *et al.* indicated that the keratinocytes in cholesteatoma may be protected against apoptosis [16].

Proteomic analysis is a useful tool for observing overall expression of protein mixtures, including body fluids, cells and tissues. It has been used in our laboratory for investigation of protein-protein interactions, development of informational database and identification of biomarkers in oral cancer and melanoma cells [17,18]. Proteomic study using 2-DE gel and MALDI-TOF mass spectrometry for the identification of potential biomarkers for cholesteatoma has been previously conducted. Proliferating cell nuclear antigen (PCNA) and osteoclast stimulating factor-1 (OSF-1) were reported to be potential biomarkers for the disease. PCNA could be correlated with cellular proliferation, and OSF-1 is possibly associated with bone destruction [19,20].

Previous studies have shown that the phosphorylation of heat shock protein 27 (HSP27) occurs at Ser-15, Ser-72 or Ser-82 in neoplastic tissues or cells [21,22]. Using 2-DE, LC-MS/MS analysis, immunohistochemistry, RT-PCR and Western blot, we, for the first time, found phosphorylation of HSP27 coupled with other regulations of cell proliferation-associated proteins in cholesteatoma. Based on proteomic findings in the current study, some potential signaling elements relevant with keratinocyte proliferation were also investigated and represented. Data from comparative examination of the proteome combined with immunological analysis in this study uncovered helpful clues for understanding the potential mechanisms of cholesteatoma growth and progression.

## 2. Results

### 2.1. 2-DE Analysis and Identification of Differential Proteins

**Figure 1:**
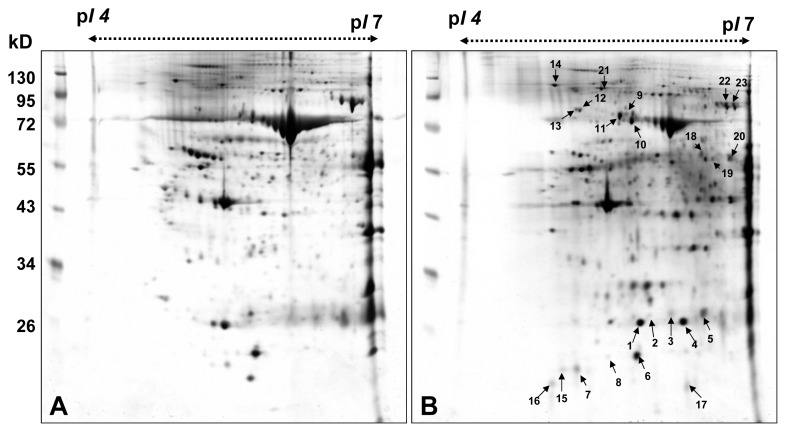
2-DE maps of (**A**) cholesteatoma tissues (pI 4–7) and (**B**) retroauricular skin (pI 4–7). A total of 23 differential proteins were identified by LC-MS/MS analysis (Table 2).

The 2-DE analysis of the samples of cholesteatoma tissues and retroauricular skin for each patient was performed for twelve patients. The proteomic data are shown in Figure 1. 2-DE analysis was run with a load of 50 μg protein on IPG 4–7, and the protein pattern was visualized by silver staining. Protein spots were detected by using PDQuest 2D image analysis software. LC-MS/MS analysis and MASCOT search identified fourteen upregulated protein spots with greater than a two-fold increase in density for cholesteatoma samples. A list of the identified proteins with their MASCOT scores, MS/MS matched peptide numbers, theoretical Molecular weight (Mw), isoelectric point (*p*I) and coverage are shown in Table 1. The five protein spots (No. 1–5) with a molecular weight of 27 kDa were identified as different species of heat shock protein 27 (HSP27). The protein spots (No. 6–8) below HSP27 were identified as peroxiredoxin-2 (PRDX2). All 2-DE maps of twelve patients are shown in Supplementary Figure 1.

### 2.2. Phosphorylation of HSP27

**Figure 2:**
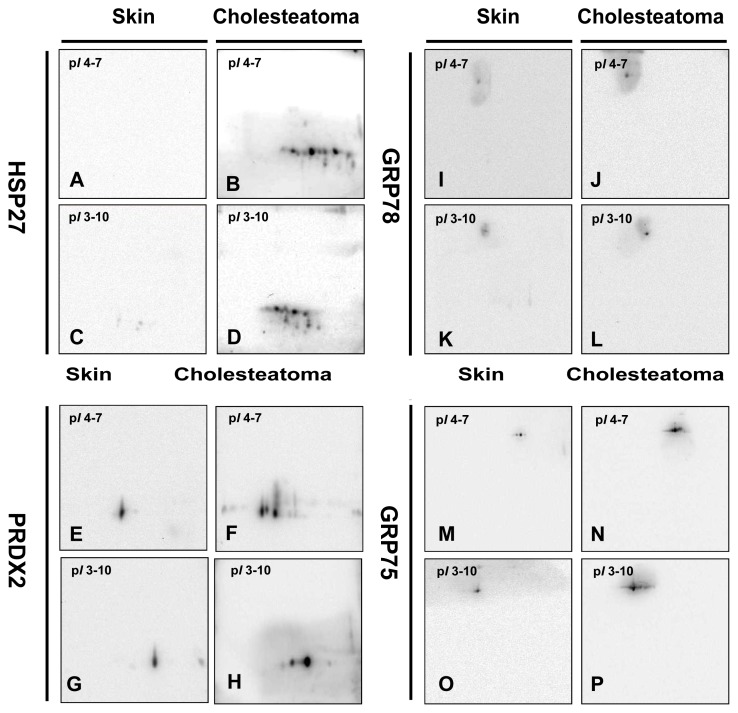
2D side-by-side comparison of the 2-DE Western blot images of HSP27, PRDX2, GRP78 and GRP75 in cholesteatoma tissues and retroauricular skin run with pI 4–7 and pI 3–10, respectively. Images **A**, **B**, **C** and **D** are for HSP27. Images **E**, **F**, **G** and **H** are for PRDX2. Images **I**, **J**, **K** and **L** are for GRP78. Images **M**, **N**, **O** and **P** are for GRP75.

**Figure 3:**
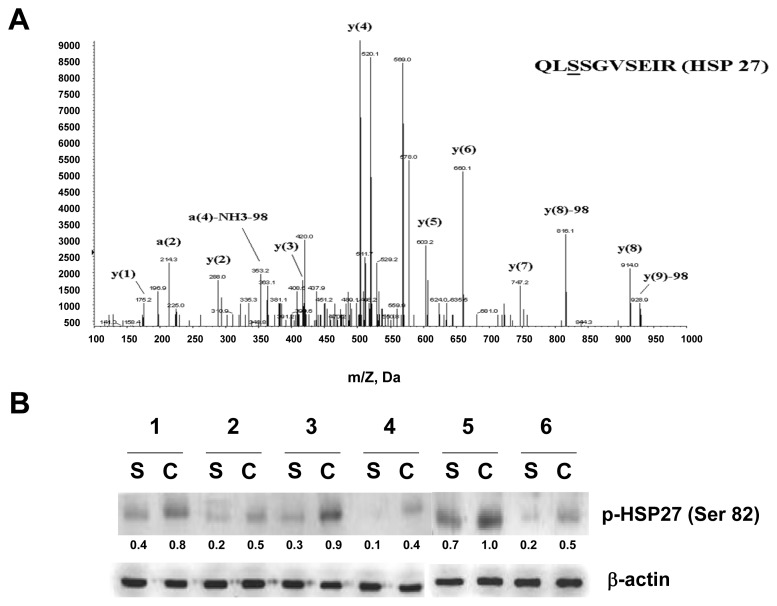
MS/MS profile and immunoblot data of phosphorylated HSP27. (**A**) MS/MS spectrum of phosphorylated HSP27 indicated the phosphorylation site at QLpSSGVSEIR; (**B**) Verification of phosphorylation-HSP27 (Ser-82) by Western blotting analysis. Cholesteatoma is presented by C, and retroauricular skin is presented by S. β-actin was used for normalization.

Five species of HSP27 have been identified on the 2-DE maps. The data suggest that the protein forms with identical molecular weight and different pI are likely caused by post-translational modification. Expression of HSP27, PRDX2, GRP78 and GRP94 in the tissues of cholesteatoma and retroauricular skin assessed by RT-PCR and Western blotting analysis is coherent with the 2-DE results. The 2D Western blot images are shown in Figure 2, and the results reinforce the suggestion that post-translational modifications caused the generation of HSP27 species with identical molecular weight and different pI. The enrichment of phosphorylation peptide by a Titansphere Phos-TiO2 kit after in-gel digestion was performed. The phosphorylation modifications of HSP27 (spot 1 and spot 3) at Ser-82 (QLpSSGVSEIR), as shown in Figure 3, were identified by QTRAP^®^ 5500Q System LC-MS/MS analysis (AB SCIEX, Framingham, USA). The signals of y_7_ and y_8_ (y_8_−98) ions on the MS/MS spectrum verify that the phosphorylation occurs at Ser-3 of this peptide (Ser-82 of HSP27).

The Western blotting analysis of phosphorylated HSP27 at Ser-82 was conducted, and the results indicate that the Ser-82 phosphorylation of HSP27 is enhanced in cholesteatoma in comparison with that in retroauricular skin (Figure 3).

### 2.3. Validation of HSP27, PRDX2, GRP75, GRP78 and GRP94 by Western Blotting Analysis and RT-PCR

**Figure 4:**
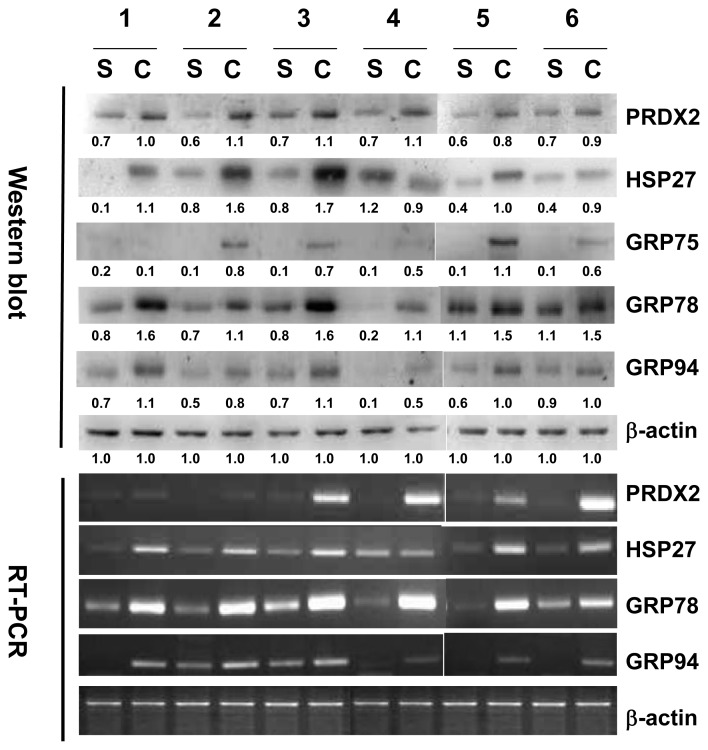
Validation of HSP27, GRP75, GRP78, GRP94 and PRDX2 by Western blotting analysis and RT-PCR. The tissues of cholesteatoma and retroauricular skin were collected from six individual patients. Cholesteatoma is presented by C, and retroauricular skin is presented by S. β-actin was used for normalization.

Changes of HSP27, PRDX2, GRP75, GRP78 and GRP94 in the tissues of cholesteatoma and the retroauricular skin of six patients were further validated by Western blotting analysis. The side-by-side comparison of HSP27, PRDX2, GRP75, GRP78 and GRP94 for each individual patient shows that these proteins are increased in cholesteatoma compared with those in retroauricular skin. The data of RT-PCR of HSP27, PRDX2, GRP75 and GRP78 also present evidence in accordance with those exhibited by Western blot (Figure 4).

### 2.4. Verification of Ras, Raf, ERK1/2, MEK1/2, p38MAPK and MAPKAPK2 by Western Blotting Analysis

**Figure 5:**
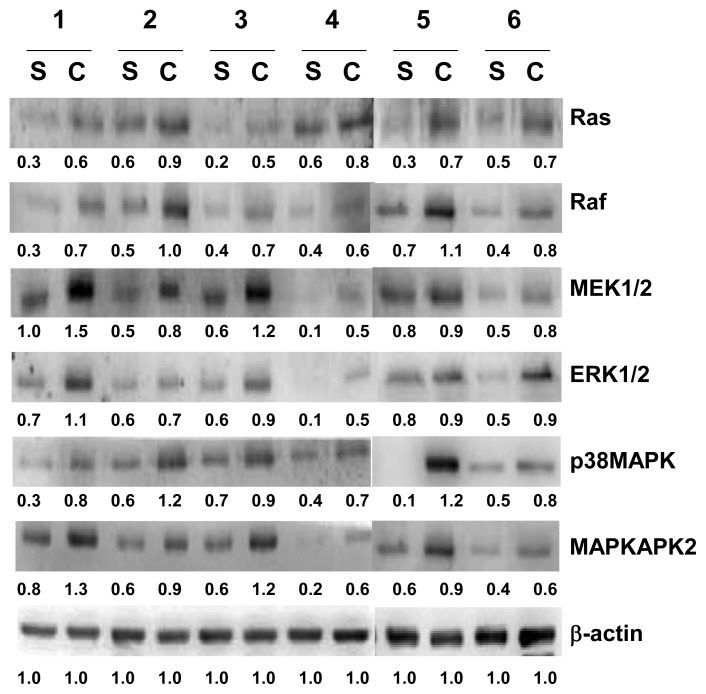
Validation of Ras, Raf, ERK1/2, MEK1/2, p38MAPK and MAPKAPK2 by Western blotting analysis. The tissues of cholesteatoma and retroauricular skin were from six individual patients. Cholesteatoma is presented by C, and retroauricular skin is presented by S. β-actin was used for normalization.

Since HSP27 could be phosphorylated in response to Vascular endothelial growth factor (VEGF) via the p38 MAPK/MAPKAPK2 pathway and triggered by the association of Epidermal growth factor (EGF) and EGF receptors (EGFR) via the Ras/Raf/ERK1/2 MAPK pathway during the progression of cholesteatoma [23], the regulation of these signaling molecules was examined. Thus, Ras, Raf, ERK1/2, MEK1/2, p38MAPK and MAPKAPK2 in the tissues of cholesteatoma and the retroauricular skin of six patients was analyzed by Western blot. The side-by-side comparison of Ras, Raf, ERK1/2, MEK1/2, p38MAPK and MAPKAPK2 for each individual patient showed that these proteins are increased in cholesteatoma in comparison with those in retroauricular skin (Figure 5).

### 2.5. Immunoreactivity of HSP27

**Figure 6:**
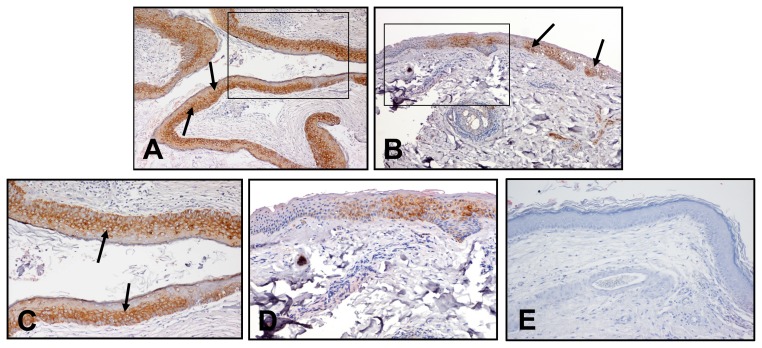
Immunoreactive staining of HSP27 for patient 5. (**A**,**C**) A strong and homogenous positive brownish staining in all layers of cholesteatoma epithelium (arrows). (**B**,**D**) Heterogeneous positive brownish staining in retroauricular skin (arrows) (**C**), (**D**). Immunoreactivity was confined to the cytoplasm of keratinocytes (arrows). The negative control was represented as (**E**). Magnification: (**A** and **B**) ×100; (**A** inset, **C**) ×200; (**B** inset, **D**) ×200; (**E**) ×200.

The immunoreactive staining of the tissues showed that HSP27 is localized in layers of cholesteatoma epithelium and retroauricular skin and confined to the cytoplasm of keratinocytes in the epithelium. The epithelium cells displayed various degrees of intensity between specimens. The staining scores between six and 12 (with a mean of 9.7) were obtained in the cholesteatomas tissues. The staining scores from two to six (with a mean of 4.0) were obtained in retroauricular skin tissues (Table 2). The immunoreactivity of HSP27 was significantly stronger in cholesteatomas than that in retroauricular skins (*p* < 0.05, Figure 6).

## 3. Discussion

In this study, the expression of HSP27, PRDX2, GRP75, GRP78 and GRP94 was found upregulated in acquired attic cholesteatoma by comparing 2-DE maps of cholesteatoma tissues with those of the retroauricular skin of the patients. These data were confirmed by Western blotting analysis. It is noteworthy that for the first time, we showed the critical upregulations of these proteomic factors and connected HSP27 phosphorylation and proteomic changes with the potential signal transduction pathways in cholesteatoma growth and progression.

Hsp70s are a family of frequently expressed heat shock proteins. Proteins with a similar structure exist in most living organisms. The Hsp70s play important roles in protein folding and help protect cells from stress [24,25]. There are three major species of Hsp70s, including HCS70 (constitutive form of heat shock protein 70), GRP75 (glucose-regulated protein 75) and GRP78 (glucose-regulated protein 78). The results of Western blotting analysis indicated that GRP75 and GRP78 in the cholesteatoma tissues are increased over retroauricular skin. It has been shown that the high density of Hsp70s may be associated with the inflammatory and immune responses in the middle ear cavity, and therefore, an essential role of HSP70 in the clinical development of cholesteatoma was suggested [26]. They may be also correlated with hyperproliferation and active differentiation of basal keratinocytes. Increased differentiation of keratinocytes could drive the programmed cell death with an increasing rate, which could lead to the accumulation of keratin debris [26]. Therefore, in the current study, elevated GRP75, GRP78 and GRP94 could be associated with chronic inflammation and granulation of attic cholesteatoma samples.

GRP78 is a chaperone protein and plays a central regulatory role in activating endoplasmic reticulum (ER) stress sensors. It regulates cellular processes, including protein assembly, as well as protein folding, and targets misfolded proteins for degradation and controls the activation of ER stress sensors [27]. The elevated expression of GRP78 could help release unfolded protein response (UPR) and maintain the balance of Ca^2+^ in ER, mitochondria and cytosol. Previous studies indicated that the induction of GRP78 maintains cellular homeostasis and prevents cells from apoptosis [28]. Suppression of GRP78 has been shown to promote apoptosis, inhibit tumor growth and enhance the cytotoxicity of chronically hypoxic cells [29–31]. Induction of GRP78 represents an important pro-survival component of ER stress, due to its anti-apoptotic properties.

GRP75 exists primarily in the mitochondria and also can be found in the cytoplasm, ER and cytoplasmic vesicles. It participates in various physiological functions, such as stress response, cell differentiation, cell proliferation, intracellular trafficking, mitochondria import, tumorigenesis and centrosome duplication [32,33]. This protein is essential in the mitochondrial import complex, which is important for translocating mitochondrial-targeted proteins [34]. It exerts tumorigenic effects through different binding partners, such as p53, intrerleukin-1 receptor type, fibroblast growth factor-1 and GRP94 [35,36]. The proliferative and tumorigenic properties of GRP75 contribute to its intracellular trafficking function and the modulation of the Ras-Raf-MAPK pathway [37,38]. Overexpression of GRP75 has been shown to lead to extended life span in nematode and normal human cells [39]. Decreased expression of GRP75 in immortalized cells causes growth arrest [40]. On the other hand, GRP94 is a member of the heat shock protein 90 family and a chaperon of the ER. It is responsible for the folding and maturation of nonglycosylated proteins. Increased expression of GRP 94 in malignant tumors has been shown to have a protective effect for tumor cells [28,41–44].

The results in the current study indicated that the expression of GRP75, GRP78 and GRP94 in the tissues of cholesteatoma is higher than that in retroauricular skin. The elevation in ER and mitochondrial stress may cause cell damage. Therefore, the upregulation of GRP75, GRP78 and GRP94 should be reasonably against the increased ER, mitochondrial stress, as well as apoptosis in attic cholesteatoma.

HSP27 is a chaperone protein of the small heat shock protein (sHsps) group. The functions of Hsps are chaperone activity, regulation of cell development, thermotolerance, inhibition of apoptosis and cell differentiation. They also participate in the signal transduction associated with apoptosis. The interaction of HSP27 with the outer mitochondrial membranes and the interference with the activation of the cytochrome c/Apaf-1/dATP complex causes the inhibition of procaspase-9. It was reported that phosphorylated HSP27 inhibits Daxx apoptotic protein and, thus, prevents the association of Daxx with Fas and Ask1 [45]. Abnormal HSP27 expression was associated with various cancers, and its tumorigenic potential has been reported in experimental models [46,47]. The dysregulation of HSP27 has been suspected as a cause for invasion and metastasis [48]; HSP27 is recognized to play the role of molecular chaperone. It is capable of modulating cell migration, cell survival, anti-proliferation, cell differentiation and vascular function through phosphorylation. HSP27 could be phosphorylated by different types of protein kinases, protein phosphatases or stimuli [49]. The study of Kindås-Mügge, using reverse transcriptase differential display polymerase chain reaction, suggested that HSP27 is a biomarker for differentiation in normal human keratinocytes [49]. The layers of epidermis consist of keratinocytes at different stages of differentiation. The highly coordinated multistep process of keratinocyte differentiation is regulated by growth factors, autocrine, paracrine, intercellular signaling mechanisms and external stimuli. Epidermal growth factor (EGF) and other growth factors could promote keratinocyte growth, differentiation and migration [50,51]. Previous studies have shown the expression of EGF and increased expression of EGF receptors (EGFR) in cholesteatoma [10]. Angiogenic growth factors (VEGF) have been reported in cholesteatoma by Sudhoff and Niwa [52,53]. The connective tissue of the perimatrix in cholesteatoma requires angioneogenesis for its growth. The wound healing process also needs angioneogenesis in response to cholesteatoma-induced tissue injury.

In this study, five protein species of HSP27 were found in cholesteatoma. This is similar to the proteomic findings in heart diseases shown by Jungblut *et al*. and Schlüter *et al.* (2009), implicating the possible biological roles of numerous HSP27 forms [54,55]. Our study for the first time discovered phosphorylation of HSP27 at Ser-82 in cholesteatoma tissues. The phosphorylation of HSP27 at Ser-82 could be induced by many different factors. It has been reported that PKC/PKD is the major pathway mediating phosphorylation of HSP27 at Ser-82 in response to VEGF [54]. It has been shown that the elevated expression of phosphorylation of p38 is in connection with involucrin, which is an end product of cell differentiation [14]. Niwa *et al*. reported that the phosphorylation of HSP27 could be induced by TNF-α or H_2_O_2_ via the p38 MAPK pathway [52]. It is therefore proposed that HSP27 is very likely phosphorylated in response to VEGF via the p38 MAPK/MAPKAPK2 pathway during the progression of cholesteatoma. This is partially verified by our immunoblotting data (Figure 6). The phosphorylated HSP27 may result in cell migration and angiogenesis. Ras protein, which plays an important role in the growth factor signal-transduction pathway, has been found in cholesteatoma specimens [56]. The accumulation of keratin debris in cholesteatoma associated with cell proliferation and differentiation of keratinocytes could be regulated by various growth factors. Investigation of UVB effects on human keratinocytes showed that EGFR and p38 MAP kinase mediate HSP27 phosphorylation [57]. The Ras/Raf/ERK1/2 MAPK signaling pathway actively involved in cholesteatoma epithelium has been reported [23]. It has been indicated that ERK1 and ERK2 are upregulated proteins involved in the MAPK pathway in cholesteatoma [14]. Moreover, HSP27 has been found to play a role in keratinocyte terminal differentiation [58]. We thus proposed that the phosphorylation of HSP27 is very likely triggered by the involvement of EGF/EGFR, the Ras/Raf/ERK1/2 pathway, as well as the MAPK pathway. In this study, we verified the changes of these crucial signaling factors by Western blot in cholesteatoma. These data in our study suggest that HSP27 together with the Ras/Raf/ERK1/2 and MAPK pathways may be relevant in stimulating keratinocyte proliferation and differentiation in cholesteatoma.

The other pathway that may induce keratinocyte proliferation and differentiation can be concluded from previous studies and the current work as the involvement of IFN-γ induction, PLC-γ, diacylglycerol (DAG), protein kinase C (PKC), PKD1/PKD2 and PRDX2 [59,60]. IFN-γ has been shown to be a potent factor to induce the expression of EGFR, as well as cell differentiation in normal neonatal skin explants or epidermal keratinocytes [61,62]. The PKC pathway and reactive oxygen stress regulate epidermal differentiation in keratinocytes [63]. PLC can activate PKC, which is important in cellular growth, differentiation and transformation; PLC-γ1 was overexpressed in cholesteatoma [64]. EGF is able to activate PLC, which is capable of activating PKC downstream through DAG. The overall processes may start with an increased EGFR expression, followed by the transductions of PLC-γ, DAG, PKC and PKD1/PKD2, as well as regulation of PRDX2. In the current study, increased expression of Peroxiredoxin2 (PRDX2) was shown by proteomic analysis and Western blot in cholesteatoma. It is worth noting that upregulation of PRDX2 has also been found in psoriasis, a hyperproliferative skin disease characterized by abnormal keratinocyte proliferation [65]. These similar findings suggest that PRDX2 may be an essential protein in the diseases or lesions correlated with keratinocyte hyperproliferation in the epidermis.

## 4. Materials and Methods

### 4.1. Materials

The Two-D Quant Kit and IPG buffer were obtained from GE Healthcare (Buckinghamshire, UK). SuperScript III and Taq DNA polymerase were from Invitrogen (Carlsbad, CA, USA). Rabbit anti-human HSP27, GRP75, GRP78, GRP94 and PRDX2 antibodies were purchased from ProteinTech Group (Chicago, IL, USA). Rabbit anti-human phosphorylation HSP27 (Ser-82), ras, raf, ERK1/2, MEK1/2, p38MAPK and MAPKAPK2 antibodies were purchased from Cell Signaling Technology (Danvers, MA, USA). Rabbit anti-human β-actin antibodies were obtained from Sigma (St. Louis, Mo, USA). Goat anti-rabbit and horseradish peroxidase conjugated IgG was from Millipore (Bellerica, MA, USA). PVDF (polyvinylidene difluoride) membranes and chemiluminescent horseradish peroxidase (HRP) substrate were from Pierce (Rockford, IL, USA).

### 4.2. Sample Preparation

Twelve patients (three males and nine females) participated in this study at the Affiliated Hospital at Kaohsiung Medical University. They were aged between 13 to 68 years, with a mean age of 40.5 years. The acquired middle ear cholesteatoma (acquired attic cholesteatoma) with slight granulation tissue specimens were resected during surgical operations. The tissues used in proteomic analysis were the epithelium of cholesteatoma after removal of granulation tissues. The retroauricular skin samples of the patients were also obtained as the controls. The protocols for using human specimens in this study were approved by the Institutional Review Board (IRB) of the hospital (approval number. KMUH-IRB-980046). Each sample (1 mm × 1 mm × 1 mm in size) was homogenized and sonicated with sample buffer (50 mM Tris-HCl; pH 8.0, EDTA); then, the sample was centrifuged at 12,000 rpm for 10 min. The supernatant was collected, and the proteins were precipitated out overnight at −20 °C by triple the volume of 10% trichloroacetic acid (TCA)/acetone solution containing 20 mM Dithiothreitol (DTT) After centrifugation at 8,000 rpm for 30 min at 4 °C, the supernatant was discarded. The pellet was rinsed three times in cold acetone containing 20 mM DTT and air-dried, then resuspended in a rehydration buffer (6 M urea, 2 M thiourea, 0.5% 3-[(3-cholamidopropyl)dimethylammonio]-1-propanesulfonate (CHAPS), 5% IPG buffer, 20 mM DTT and 0.002% bromophenol blue) at 4 °C overnight. The protein contents were determined using a 2-D Quant Kit (GE Healthcare).

### 4.3. Two-Dimensional Gel Electrophoresis

The first dimension electrophoresis (isoelectric focusing) was performed on a GE Healthcare Ettan IPGphor 3 with the protocol described previously [66]. Proteins (50 μg) extracted from whole tissue were loaded on 11 cm Immobilized pH gradient (IPG) strips for Isoelectric focusing (IEF) and then were separated on SDS-PAGE (12.5%).

### 4.4. Protein Spot Identification by LC-MS/MS

#### 4.4.1. In-Gel Digestion

Spots of interest were excised into a piece of 1 mm × 1 mm, then placed in a microcentrifuge tube. Briefly, 25 mM ammonium bicarbonate (pH 8.5) was added to the tube, which was shaken at 37 °C for 1 h. The gel piece was then dehydrated in acetonitrile and dried by SpeedVac to remove the remaining acetonitrile. Zero-point-one micrograms of trypsin in 10 μL 25 mM ammonium bicarbonate (pH 8.5) was added to the gel piece. Protein digestion was run overnight at 37 °C. Fifty microliters of 5% trifluoroacetic acid (TFA) in 50% acetonitrile was added to quench the trypsin digestion. Peptides were extracted with 25 mM ammonium bicarbonate, 50% acetonitrile and 0.1% trifluoroacetic acid. The peptide solution was concentrated for the following LC-MS/MS analysis.

#### 4.4.2. LC-MS/MS Analysis and MASCOT Database Searching

After desalting with a ZIPTip^®^_C18_ (Millipore, Bellerica, MA, USA), the resulting peptide mixture was separated using a NanoLC 1200 System (Agilent) utilizing a Zobax column (2.1 mm × 150 mm) packed with 3 μm C18 particles (Agilent, Santa Clara, CA, USA) with a linear gradient from 5% to 60% acetonitrile containing 0.1% formic acid over 60 min. The separated peptides were analyzed online on a QTrap 5500 mass spectrometer (AB SCIEX, Framingham, USA) equipped with a nano ESI source. The scan range was from *m*/*z* 100 to 1,000 for MS and MS/MS. The raw data were processed into a text file format of WIFF with Analyst 1.5.1, and the resulting text file was searched using the MASCOT search engine v2.2 (Matrix Science, Boston, USA) with the following search parameters: (1) the protein database was set to be Swiss-Prot; (2) the taxonomy was set as *Homo sapiens* (human); (3) one trypsin missed cleavage was allowed; (4) the mass tolerance was set at 1.5 Da for the precursor and 0.8 Da for the product ions; (5) carbamidomethyl (C) was chosen for fixed modification; (6) oxidation (M), phospho- (ST) and phosphor- (Y) were chosen for variable modifications; and (7) proteins with scores above the significance threshold (*p* < 0.05) were shown as significant hits. The hit with the highest score that contained at least two peptides with scores beyond the identity threshold was regarded as the identified protein from each gel spot. All MS/MS spectra of the identified peptides were further verified by manual interpretation.

### 4.5. Western Blotting Analysis

After 1-DE and 2-DE PAGE analysis of the samples collected from the patients, the proteins on gel were transferred to a PVDF membrane (Millipore, MA, USA), for 1.5 h at 400 mA using a Transphor TE 62 (Hoeffer, Holliston, MA). The membranes were then incubated with HSP27, phosphorylation HSP27, GRP75, GRP78, GRP94 and β-actin antibodies at 4 °C for 2 h or overnight. The membranes were washed three times in PBST (10 mM NaH_2_PO_4_, 130 mM NaCl, 0.05% Tween 20), then probed with the second antibodies (goat anti-rabbit and horseradish peroxidase conjugate (1:5,000) in blocking solution) for 1 h. After washing with PBST three times, the enzyme activity on the blot was visualized through chemiluminescence by adding ECL Western Blotting Reagents (Pierce Rockford, IL, USA).

### 4.6. RNA Isolation and RT-PCR

Total RNA was isolated from both cholesteatoma and normal retroauricular skin samples using TRIzol reagent RNA Extraction Kits (Qiagen, Hilden, Germany). The RNA concentrations were measured using a GeneQuant 1300 spectrophotometer (GE Healthcare, Buckinghamshire, UK). Reverse transcription was carried out in the reaction containing RNA samples, dNTP, random primers, 5× first strand buffer, DTT (0.1 M) and SuperScript III (Invitrogen, Carlsbad, CA, USA) on a PCR machine (Bio-Rad, Hercules, CA, USA). The sequences of primers used in the PCR reactions are as below:

(1) β-actin follows:
  5′-3′AGAGATGGCCACGGCTGCTT (forward);
  5′-3′ATTTGCGGTGGACGATGGAG (reverse).
(2) Heat shock protein 27 (HSP27) follows:
  5′-3′ ACGAGCATGGCTACATCTCC (forward);
  5′-3′ CTTTACTTGGCGGCAGTCTC (reverse).
(3) Thioredoxin peroxidase 2 (PRDX2) follows:
  5′-3′GTGTCCTTCGCCAGATCACT (forward);
  5′-3′ ACGTTGGGCTTAATCGTGTC (reverse).
(4) Glucose-regulated protein 78 (GRP78) follows:
  5′-3′ TCCTATGTCGCCTTCACT (forward);
  5′-3′ ACAGACGGGTCATTCCAC (reverse).
(5) Glucose-regulated protein 94 (GRP94) follows:
  5′-3′ GGGAGGTCACCTTCAAGTCG (forward);
  5′-3′ GGGTGTAGACGTGGAGCTC (reverse).

For PCR, the reaction tubes containing 10× buffer, MgCl_2_, dNTPs, Taq DNA polymerase (Invitrogen, Grand Island, NY, USA) and each of the forward and reverse primers were preheated at 95 °C for 3 min. The three stages of 30 cycles of PCR were accomplished as follows: denaturation at 95 °C for 30 s, annealing at 55 °C for 30 s, elongation at 72 °C for 30 s and extension was completed at 72 °C for 10 min. The PCR products were electrophoresed on 1.5% agarose gel.

### 4.7. Immunohistochemical Staining of Hsp27

The samples from the 12 patients were then assessed by immunohistochemistry. A cholesteatomas specimen and a retroauricular skin specimen of each patient were resected during surgical operations. Immunohistochemistry was performed on 4 μm thick paraffin sections. Paraffin sections of all samples were de-paraffinized, rehydrated and autoclave-treated at 121 °C for 10 min in DAKO Target Retrieval Solution, pH 6.0 (DAKO, Glostrup, Denmark), to induce antigen retrieval. Endogenous peroxidase in the section was blocked by incubation in 3% hydrogen peroxide for 5 min. The sections were incubated with HSP27 primary antibodies (1:60; Leica Novocastra, Newcastle upon Tyne, UK) at room temperature for 1 h. Then, the DAKO REAL EnVision Detection kit (DAKO) was applied for 30 min. Finally, sections were incubated in 3′3-diaminobenzidine for 5 min, followed by Mayer’s hematoxylin counterstaining and mounting. Negative controls were obtained by replacing the primary antibody with non-immune serum.

The percentage of immunoreactive staining for HSP27 in all 24 samples was evaluated by two independent observers. They were assigned a score number according to the following rules: a score of 0 for 0% epithelium cells positive, a score of 1 for 1%–24% epithelium cells positive, a score of 2 for 25%–49% epithelium cells positive, a score of 3 for 50%–74% epithelium cells positive and score of 4 for 75%–100% epithelium cells positive. The intensity of cellular staining was also assigned a score number: a score of 0 for zero intensity, a score of 1 for weak intensity, a score of 2 for moderate intensity and a score of 3 for strong intensity. A staining score was obtained by multiplying the percentage score with the intensity score, with a maximum score of 12. Statistical evaluations were performed using the paired *t*-test. A difference considered statistically significance is *p* < 0.05.

## 5. Conclusions

Taken together, in the current study, HSP27 and PRDX2 were enhanced, and phosphorylation of HSP27 at Ser-82 was identified in acquired attic cholesteatoma tissue. Furthermore, phosphorylation of HSP27 at Ser-82 can be mediated through the PKC/PKD pathway in response to VEGF or via the p38 MAPK pathway in cholesteatoma. The phosphorylated HSP27 could be associated with the activation of cell migration, angiogenesis and proliferation in epithelial cells, resulting in subsequent growth of cholesteatoma. Our data implicate that the phosphorylation of HSP27 is probably induced by the pathway, including EGF/EGFR, Ras/Raf/MEK1/2/ERK1/2, as well as p38 MAPK. Upregulation of PRDX2 could be relevant with keratinocyte hyperproliferation in the epidermis, and PRDX2 elevation is possibly mediated via EGFR, PLC-γ, DAG, PKC and PKD1/PKD2 in the other pathway. In addition, upregulation of GRP75, GRP78 and GRP94 may not only be associated with chronic inflammation of attic cholesteatoma, but also counter ER and mitochondria stresses, reduce cell apoptosis, prevent protein unfolding and could also favor keratinocyte proliferation in attic cholesteatoma. These results shed light on the potential mechanisms of signal transduction in acquired middle ear cholesteatoma and are helpful for understanding the pathogenesis of cholesteatoma.

## Figures and Tables

**Table 1:** Summary of differential proteins identified by LC-MS/MS analysis.

Spot No.	Protein name	Accession No.	Calculate Mw/*p*I	Peptide matched	Sequence covered %	MASCO T score	Biological process
1	Heat-shock protein 27	P04792	22.7/5.98	41	61	547	negative regulation of apoptotic process positive regulation of angiogenesis positive regulation of endothelial cell chemotaxis y positive regulation of interleukin-1 beta production positive regulation of tumor necrosis factor biosynthetic process regulation of I-kappaB kinase/NF-kappaB cascade regulation of translational initiation response to unfolded protein
2	Heat-shock protein 27	P04792	22.7/5.98	10	30	190	
3	Heat-shock protein 27	P04792	22.7/5.98	9	25	140	
4	Heat-shock protein 27	P04792	22.7/5.98	36	48	385	
5	Heat-shock protein 27	P04792	22.7/5.98	10	37	150	

6	Peroxiredoxin-2	P32119	21.8/5.66	19	43	174	hydrogen peroxide catabolic process
7	Peroxiredoxin-2	P32119	21.8/5.66	40	44	641	negative regulation of apoptotic process
8	Peroxiredoxin-2	P32119	21.8/5.66	26	42	336	removal of superoxide radicals

9	75 kDa glucose-regulated protein	P38646	73.6/5.87	13	15	157	negative regulation of apoptotic process protein export from nucleus protein folding protein targeting to mitochondrion

10	Heat shock cognate 71 kDa protein	P11142	70.8/5.37	39	34	339	mRNA metabolic process negative regulation of transcription protein folding regulation of cell cycle response to unfolded protein
11	Heat shock cognate 71 kDa protein	P11142	70.8/5.37	59	46	624	

12	78 kDa glucose-regulated protein	P11021	72.2/5.07	15	16	123	ER overload response ER-associated protein catabolic process negative regulation of apoptotic process positive regulation of protein ubiquitination regulation of protein folding in endoplasmic reticulum
13	78 kDa glucose-regulated protein	P11021	72.2/5.07	55	42	719	

14	94 kDa glucose-regulated protein	P14625	92.4/4.76	75	37	799	ER-associated protein catabolic process actin rod assembly activation of signaling protein activity involved in unfolded protein response negative regulation of apoptotic process protein folding

15	Uncharacterize d protein C7orf24	O75223	20.9/5.07	13	26	158	glutathione biosynthetic process release of cytochrome c from mitochondria
16	Uncharacterize d protein C7orf24	O75223	20.9/5.07	7	18	104	

17	NEDD8-conjugating enzyme Ubc12	P61081	20.8/7.57	3	12	35	protein neddylation

18	Serum albumin precursor	P02768	69.3/5.92	41	25	388	lipoprotein metabolic process maintenance of mitochondrion location negative regulation of apoptotic process response to nutrient

19	Glial fibrillary acidic protein	P14136	49.8/5.42	11	6	141	extracellular matrix organization intermediate filament organization response to wounding

20	Ig alpha-1 chain C region	P01876	37.6/6.08	6	9	81	immune response protein-chromophore linkage

21	Transitional endoplasmic reticulum ATPase	P55072	89.2/5.14	27	21	176	ER-associated protein catabolic process activity involved in apoptotic process double-strand break repair endoplasmic reticulum unfolded protein response protein ubiquitination

22	Serotransferrin precursor	P02787	77.0/6.81	31	23	306	cellular iron ion homeostasis transferrin transport transmembrane transport
23	Serotransferrin precursor	P02787	77.0/6.81	20	18	181	

**Table 2:** Statistical characteristics of patients with cholesteatoma and the staining scores of HSP27 for the twelve patients.

Patient No.	Sex	Age	Hsp27 staining score	
			
			Cholesteatoma a	Retroauricular skin b
1	Male	22	12	4
2	Female	13	12	6
3	Female	56	12	6
4	Female	68	6	3
5	Female	31	12	6
6	Male	35	9	2
7	Female	58	9	2
8	Male	50	8	3
9	Female	57	8	2
10	Female	26	9	4
11	Female	14	12	6
12	Female	57	8	3

Mean		40.5	9.7	4.0

Paired *t*-test:

a b *p* < 0.05.
